# Clinico-Serological Profile of Total IgE Levels Detected by Enzyme-Linked Fluorescent Assay (ELFA) Among Pediatric Patients With Allergic Conditions

**DOI:** 10.7759/cureus.110272

**Published:** 2026-06-04

**Authors:** Supriya B Gachinmath, Dhyan K Murthy, Asmita Parida

**Affiliations:** 1 Microbiology, St. John's Medical College, Bengaluru, IND; 2 Life Sciences, CHRIST (Deemed to be) University, Bengaluru, IND

**Keywords:** allergic diseases, asthma, children, elfa method, ig e levels

## Abstract

Background and objective: The global rate of existence for pediatric allergies is increasing; the importance of IgE as the predominant immune component involved in allergic disease (or type 1 hypersensitivity) is acknowledged throughout the world. Total serum IgE levels are widely used to evaluate patients with atopic diseases, but references correlating IgE levels determined by enzyme-linked fluorescent assay (ELFA) with clinical symptoms are limited in the pediatric population. This study aimed to analyze both the clinical and serological characteristics of total IgE levels in patients with suspected allergy.

Materials and methods: A cross-sectional study of 237 pediatric patients (aged zero to 18 years old) was performed at a tertiary care center in Bengaluru, Karnataka, India, with an automated ELFA (VIDAS; bioMérieux, Marcy-l'Étoile, FRA) to measure total serum IgE. Demographic data and clinical characteristics were recorded. The chi-square test analyzed the association between age, sex, and elevated IgE levels. Lastly, Spearman's rank correlation examined the relationship between eosinophils and IgE.

Results: There was a statistically significant association between elevated serum IgE levels (>100 kIU/L) and age, with the highest prevalence found among the 11 to 15 years age group (p=0.0049). The common IgE-associated diagnoses were asthma (20.1%), n=32, and allergic rhinitis (AR) (18.2%), n=29. The majority of patients had IgE levels between 100 and 1000 kIU/L. There was a statistically significant, positive correlation between IgE levels and absolute eosinophil count (AEC) (rho=0.249; p=0.008). Males displayed a greater frequency of elevated IgE levels, but this relationship was not statistically significant.

Conclusion: Total serum IgE estimation by ELFA is a reliable adjunctive tool for diagnosis and monitoring of pediatric allergic diseases, particularly respiratory conditions, atopic conditions, immunological disorders, and correlation of elevated IgE levels with eosinophilic inflammation.

## Introduction

In the last few decades, there has been a significant increase in the number of children with allergic diseases, with a growing prevalence of these diseases across the globe; children are now more likely to report having at least one allergy than ever before [1]. The allergic disorders in pediatric populations include allergic rhinitis (AR), asthma, eczema, and food allergies. There is an increasing incidence of these diseases in developing countries, which is believed to be related to factors including environmental exposures, lifestyle changes, dietary changes, and the hygiene hypothesis [2].

The role of IgE in the pathophysiology of all allergy-related conditions is dominant, as IgE mediates the type one hypersensitivity reaction via its adhesion to mast cell and basophil high-affinity IgE receptors [3]. Measurement of the total levels of serum IgE has emerged as a valuable tool for diagnostic purposes in clinical allergy [4]. An elevated level of serum IgE is associated with the increased likelihood of developing an allergic reaction and correlates with the degree of illness severity for multiple atopic conditions [5].

Elevated IgE is an important laboratory finding in certain primary immunodeficiencies, particularly hyper-IgE syndrome, Wiskott-Aldrich syndrome, Omenn syndrome, and atypical complete DiGeorge syndrome [6]. Limited studies have evaluated primary immunodeficiency in patients with serum IgE ≥2000 IU/mL. Allergic patients frequently show elevated antigen-specific and total IgE levels (1,000-10,000 IU/mL) [7], though markedly elevated levels are less frequent in AR, asthma, and atopic dermatitis [8]. The IgE production depends on IL-4-mediated TH2 responses, leading to mast cell activation and hypersensitivity reactions [9]. Elevated IgE may also occur in helminth infections, multiple myeloma, and autoimmune diseases [10].

Recent evidence suggests that elevated IgE levels are associated with chronic inflammatory conditions, including allograft rejection and atherosclerosis [11]. Total IgE levels above 1000 IU/mL have been reported in various atopic conditions such as food allergy, bronchopulmonary mycosis, and asthma [12]. Asthmatic patients with high IgE are at increased risk of complications like airway infections and rhinosinusitis. Markedly elevated IgE is less common in helminth infections and atopic dermatitis. Non-allergic causes, including hyper-IgE syndrome with signal transducer and activator of transcription 3 gene (STAT3) mutations, should also be considered [13]. Increased IgE has additionally been observed in autoimmune diseases such as systemic lupus erythematosus [14].

Respiratory allergies affect all age groups, with allergen patterns influenced by geography, climate, and socioeconomic factors. Total serum IgE and absolute eosinophil count (AEC) are commonly used markers in evaluation. This study aimed to assess allergen sensitivity in patients with AR and/or asthma and correlate skin prick test reactivity with total IgE and AEC [15]. Although total IgE testing is routine, few pediatric studies have analyzed serum IgE using ELFA in relation to clinical phenotype [16]. The study includes infants to adolescents (<1 year, one to 18 years) diagnosed at St. John’s Medical College Hospital (Bengaluru, KA, IND), from both urban and rural backgrounds, encompassing all allergy-related conditions.

## Materials and methods

Study design and sample type

This cross-sectional study assessed total IgE levels in allergic children by using the ELFA, a highly sensitive and rapid automated procedure [17]. Blood samples were obtained from patients and handled in accordance with standard laboratory procedures (i.e., processed, centrifuged, and stored). The IgE results were reported in kIU/L based on criteria set forth by both the manufacturer and laboratory quality control protocols, along with clinical and demographic data relevant for analysis. Only pediatric patients (aged 18 years or younger) were included in this study. This study was approved by the Institutional Ethics Committee of St. John's Medical College Hospital (approval no. 347/2025).

Sample collection and preparation

Before testing, reagents were allowed to come to room temperature (~30 minutes) after being removed from refrigeration. Samples, calibrator (duplicate runs of S1), and control (C1) were processed with one IgE reagent strip and one IgE surface plasmon resonance (SPR), while resealing storage packs immediately after removing reagents. A test volume of 100 uL was utilized, and the SPR and strip placements were in accordance with the required assay identification. Following pipetting, reagents were returned to their original temperature (2-8 °C). The assay was carried out on the VIDAS (bioMérieux, Marcy-l'Étoile, FRA) automated ELFA analyzer in roughly 30 minutes. The VIDAS total IgE kit contains one anti-human IgE SPR, reagent strips, R1 (IgE diluent), S1 (calibrator), C1 (positive control), and a master lot entry card for lot-based calibrations and quality assurance. Data were entered into Microsoft Excel (Microsoft Corp., Redmond, WA, USA) and analyzed with the GraphPad online tool (GraphPad Software Inc., San Diego, CA, USA).

Result calculation and interpretation

**Figure 1:**
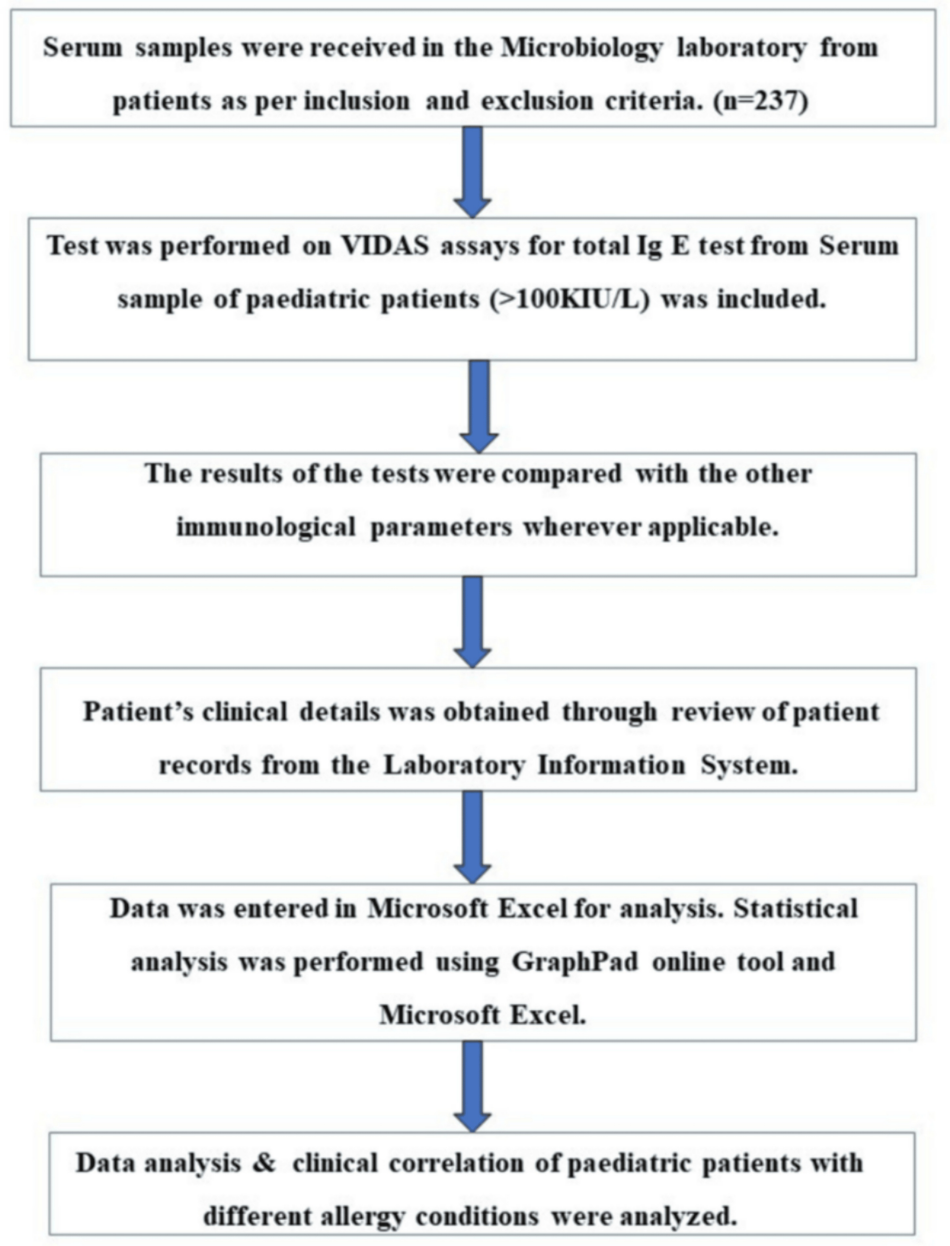
Flowchart representing sample collection, processing, and analysis

The investigation utilized the instrument's built-in computer to analyze results instantaneously upon assay completion. Measurement of fluorescence for each sample was accomplished through two readings. The first was taken when there was a background fluorescence measurement of the substrate cuvette prior to adding SPR, and the second occurred when there was an actual measurement of fluorescence after incubation, at which time the enzyme within the SPR had reacted with the substrate. Relative fluorescence value (RFV) was calculated by subtracting background fluorescence from post-incubation fluorescence for each sample and then reported on the results sheet. The IgE concentration of each sample was determined from a four-parameter logistic (4PL) standard curve using stored specimen records and retrieved in kIU/L according to the International Organization for Standardization (ISO) standard (2nd IRP 75/502). Numbers greater than 1,000 kIU/L were reassessed after dilution (1:10 or 1:100), where appropriate, and the resulting value was computed by applying the identifying dilution factor. Interpretation of results was performed in conjunction with clinical history, additional laboratory testing, and local hospital laboratory records (Figure 1).

Statistical analysis

Statistical analysis of the data was conducted via SPSS Statistics version 25 (IBM Corp., Armonk, NY, USA). Categorical variables were summarized using frequencies and percentages. The association between resistance patterns and relevant variables was evaluated using the chi-square test or Fisher’s exact test, depending on the expected cell counts. A p-value < 0.05 was considered statistically significant. The graphs were created using RStudio version 2026.01.0 "Apple Blossom" (RStudio, Boston, MA, USA).

## Results

A total of 237 pediatric patients were included in the study. The majority belonged to the 11-to-15-year age group (29.5%), followed by the six-to-10-year age group (26.6%). Males accounted for 56.5% of the study population, with a male-to-female ratio of approximately 1.4:1. Elevated serum IgE levels (>100 kIU/L) were observed in 67.1% of participants. Respiratory allergic conditions, particularly asthma and AR, were the most common clinical diagnoses among IgE-positive patients (Table 1).

**Table 1:** Baseline demographic characteristics of the study population The values are presented as n(%) unless otherwise specified. Some patients had more than one diagnosis. AR: Allergic rhinitis; ABPA: Allergic bronchopulmonary aspergillosis; AEC: Absolute eosinophil count

Characteristics		All patients (n=237)	IgE >100 kIU/L (n=160)
Age distribution, n (%)	0-5 years	59 (24.9%)	29 (49.1%)
	6-10 years	63 (26.6%)	44 (69.8%)
	11-15 years	70 (29.5%)	54 (77.1%)
	16-18 years	45 (19.0%)	33 (73.3%)
Sex, n (%)	Male	134 (56.5%)	93 (58.1%)
	Female	103 (43.5%)	67 (41.9%)
	Male:female ratio	1.3:1	1.4:1
Clinical diagnoses (comorbidities), n (%)			Mean IgE (kIU/L)
	Asthma	32 (20.1%)	1161
	AR	29 (18.2%)	1001
	Allergic bronchopulmonary aspergillosis (ABPA)	24 (15.1%)	1669
	Bronchiectasis	15 (9.4%)	1168
	Urticaria	14 (8.8%)	d902
	Chronic lung disease	10 (6.3%)	1714
	Eczema	10 (6.3%)	1505
	Hyper-IgE syndrome	8 (5.0%)	3436
	Immunodeficiency disorder	8 (5.0%)	2234
	Dermatitis	7 (4.4%)	1771
Confounding variables	Elevated AEC	—	Weak positive correlation with IgE (ρ=0.249, p=0.008)
	Inpatient vs. outpatient setting	—	OPD: 61%; Pediatric ward: 20.8%; Others: 18.2%
	Pediatric age range (all patients)	0-18 years	0-18 years

Age-wise distribution of patients with serum IgE levels >100 kIU/L

**Figure 2:**
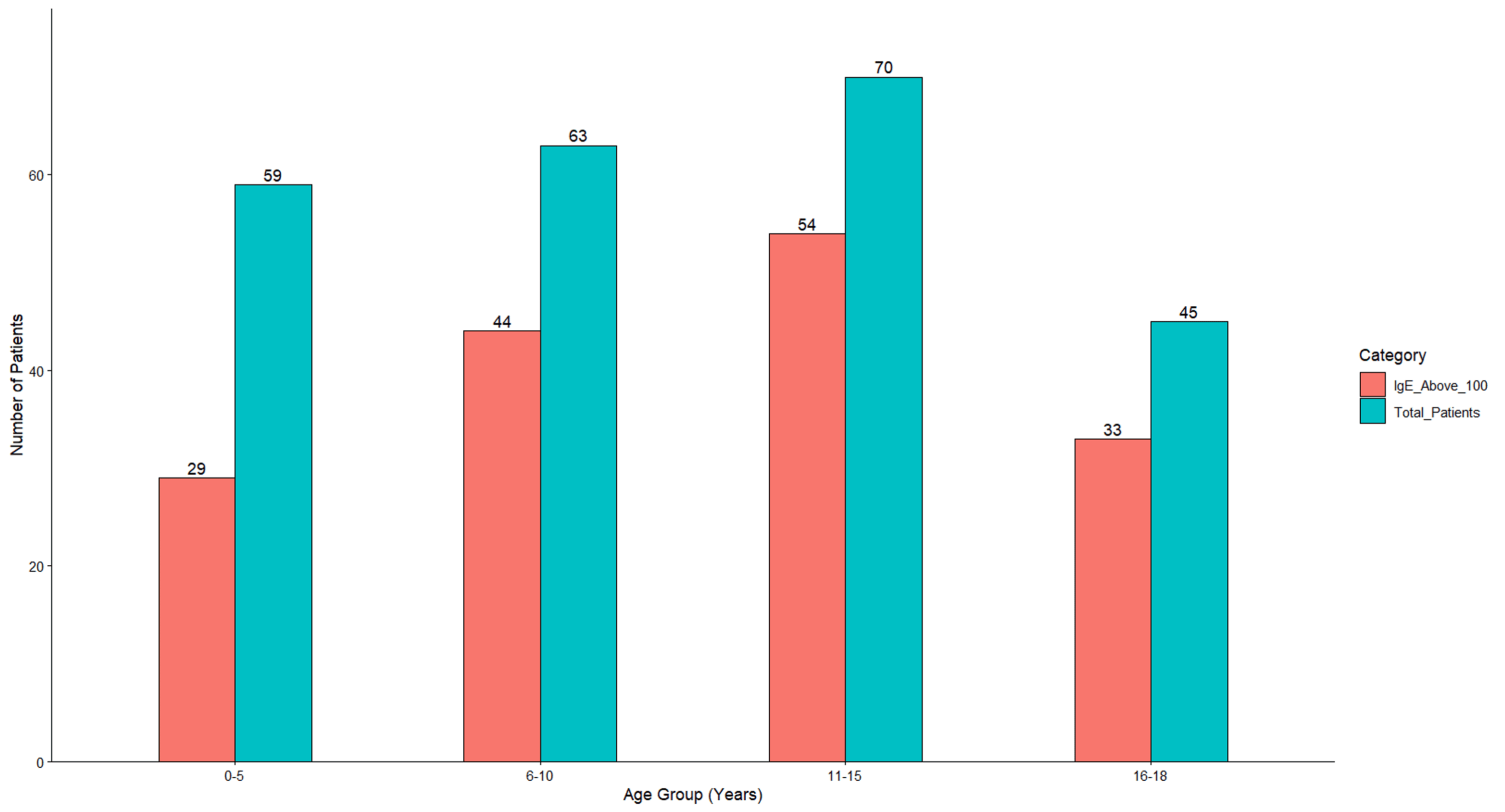
Age-wise distribution of patients with serum IgE levels >100 kIU/L

Figure 2 and Table 2 depict the various age groups associated with patients assessed to be within and over a total serum IgE of >100 kIU/L. The total number of patients fell within four specified age categories (zero to five, six to 10, 11 to 15, and 16 to 18 years). The largest number of patients included in the assessment were 11-15 years old with a positivity of 77.1%, followed by those aged six to 10 years old with a positivity of 69.8%, those aged zero to five years old with a positivity of 49.1%, and 16 to 18 years old with a positivity of 73.3%. The highest number of patients with elevated serum IgE levels was in the 11-15-year age group (n=54/70), followed by the 16-18-year age group (n=33/45). The higher proportion of patients with elevated levels increased within each respective group, as indicated by the 11-15 years age group, with an increase in proportion to 77.1%, and remained high during the adolescent age groups.

**Table 2:** Age-wise distribution of patients with elevated serum IgE levels (n = 237)

Age group (years)	Total patients, n (%)	IgE >100 kIU/L, n (%)
0-5	59 (24.9%)	29 (49.1%)
6-10	63 (26.6%)	44 (69.8%)
11-15	70 (29.5%)	54 (77.1%)
16-18	45 (19.0%)	33 (73.3%)
Total	237 (100%)	160 (67.5%)

The association between age group and elevated serum IgE levels was assessed using the chi-square (χ²) test. The calculated χ² was found to be 12.88, and the critical χ² (0.05) is 7.815. The degrees of freedom (df) were calculated as 3. Since the calculated χ² value (12.88) is greater than the tabulated value at 0.05 significance (7.815), the result is statistically significant as p<0.05 (p=0.0049). Therefore, the null hypothesis is rejected, and we accept the alternative hypothesis. There is a statistically significant association between age group and serum IgE levels, with higher age groups showing a greater prevalence of elevated IgE levels, especially in the age group from 11 to 16 years.

Sex-wise distribution of patients across the elevated IgE ranges

**Figure 3:**
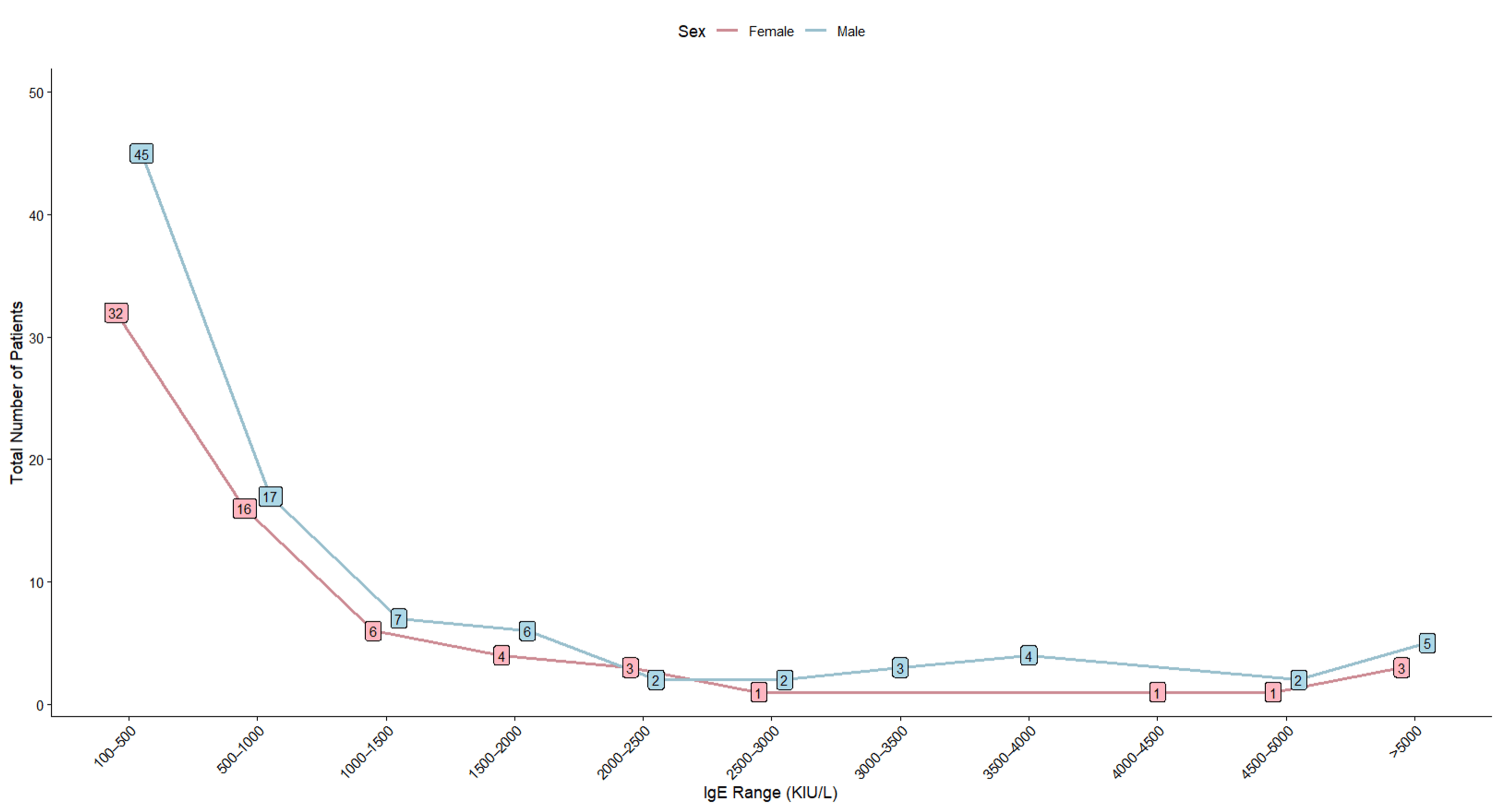
Sex-wise distribution among total number of patients across elevated IgE ranges

Among the 237 pediatric patients included in the study, 160 (67.5%) demonstrated elevated serum IgE levels (>100 kIU/L). The distribution of IgE-positive patients across different IgE concentration ranges was further analyzed according to gender. Overall, males constituted 93 (58.1%) of the IgE-positive cohort, while females accounted for 67 (41.9%), showing a relative male predominance. The majority of patients were clustered within the lower IgE range of 100-500 kIU/L, with progressively fewer patients observed as IgE levels increased. A noticeable male predominance was evident in the higher IgE categories (>3000 kIU/L) (Table 3 and Figure 3).

**Table 3:** Gender-wise distribution of patients across serum IgE ranges (n = 161)

IgE range (kIU/L)	Male, n (%)	Female, n (%)	Total, n (%)
100-500	45 (48.4%)	32 (47.8%)	77 (48.1%)
500-1000	17 (18.3%)	16 (23.9%)	33 (20.6%)
1000-1500	7 (7.5%)	6 (9.0%)	13 (8.1%)
1500-2000	6 (6.5%)	4 (6.0%)	10 (6.3%)
2000-2500	2 (2.2%)	3 (4.5%)	5 (3.1%)
2500-3000	2 (2.2%)	1 (1.5%)	3 (1.9%)
3000-3500	3 (3.2%)	0 (0%)	3 (1.9%)
3500-4000	4 (4.3%)	0 (0%)	4 (2.5%)
4000-4500	0 (0%)	1 (1.5%)	1 (0.6%)
4500-5000	2 (2.2%)	1 (1.5%)	3 (1.9%)
>5000	5 (5.4%)	3 (4.5%)	8 (5.0%)
Total	93 (58.1%)	67 (41.9%)	160 (100%)

Distribution of IgE-positive patients across hospital wards (pediatrics)

**Figure 4:**
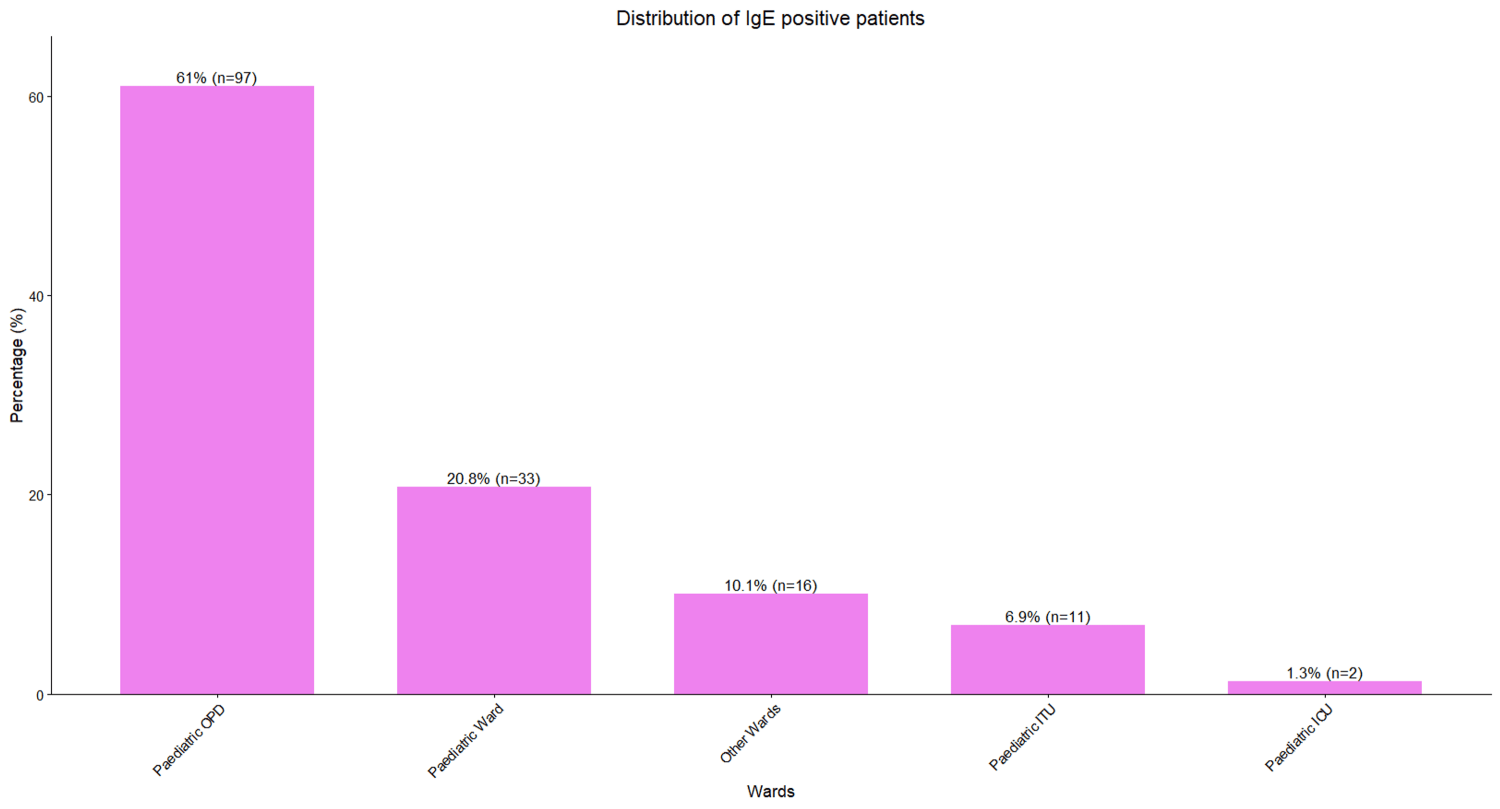
Distribution of IgE positive patients across different pediatric wards

The highest proportion of IgE-positive patients was seen in the pediatric OPD (61%). Among inpatient departments, pediatric wards were 20.8%, followed by other wards such as the dermatology OPD, immunology/dermatology common ward, and the ENT OPD with 10.1%; the pediatric intensive treatment unit (ITU) with 6.9%; and the pediatric ICU with 1.3%, showing the lowest percentages, indicating fewer critically ill patients (Table 4 and Figure 4).

**Table 4:** Distribution of IgE-positive patients across hospital wards (n = 160) ITU: Intensive treatment unit

Ward	No. of patients, n (%)
Paediatric OPD	97 (60.6%)
Paediatric Ward	33 (20.6%)
Other Wards*	16 (10.0%)
Paediatric ITU	11 (6.9%)
Paediatric ICU	2 (1.3%)
Total	160 (100%)

Distribution of clinical diagnoses among patients with elevated serum IgE levels and their corresponding mean IgE concentrations

**Figure 5:**
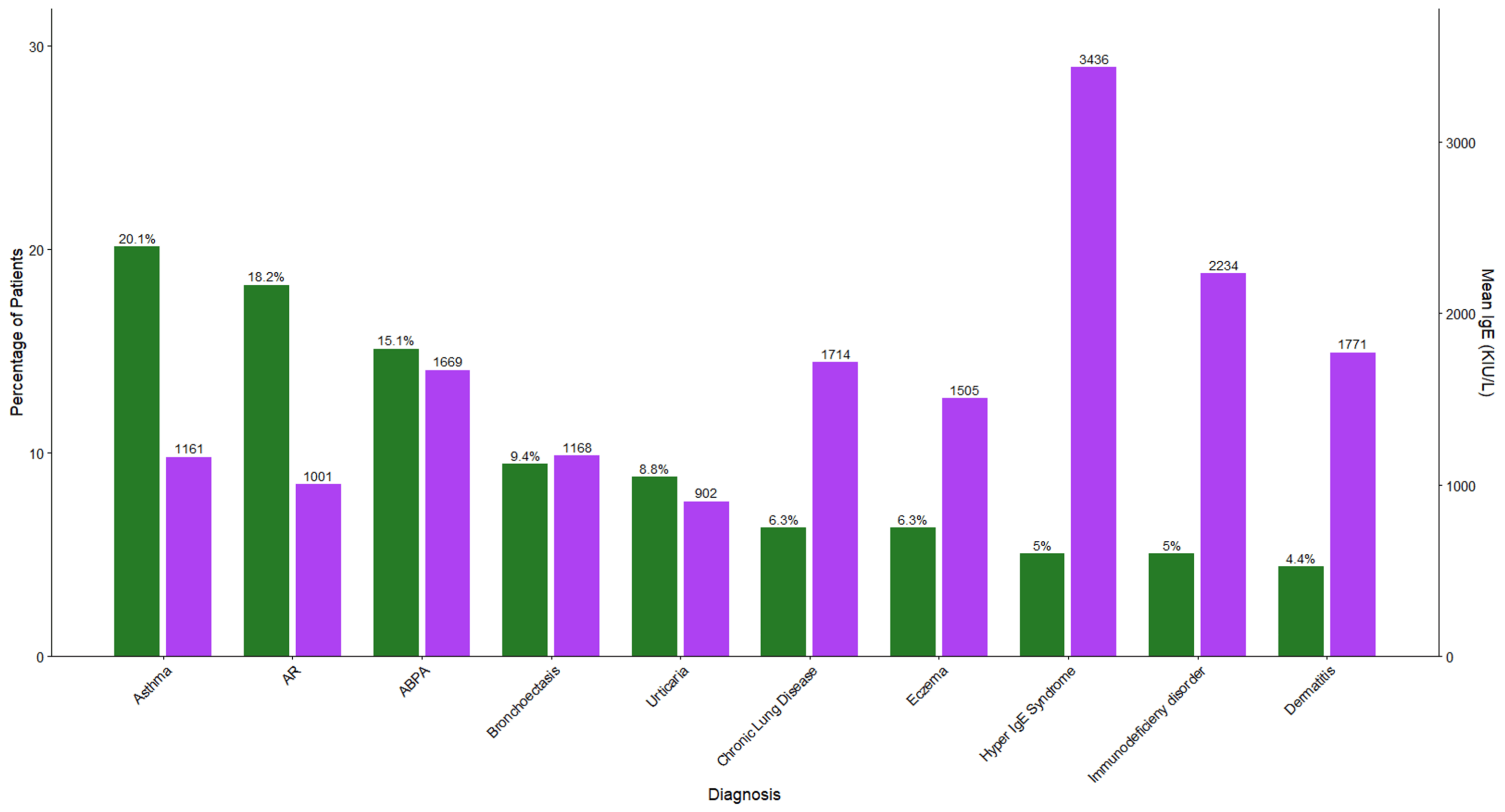
Distribution of clinical diagnoses among patients with elevated serum IgE levels, showing the percentage of patients and corresponding mean serum IgE concentrations in kIU/L. ABPA: Allergic bronchopulmonary aspergillosis

A total of 160 patients were included in the study. The most common diagnosis was asthma, accounting for 20.1% of patients (n = 32), followed by AR in 18.2% (n = 29) and allergic bronchopulmonary aspergillosis (ABPA) in 15.1% (n = 24). Bronchiectasis and urticaria were identified in 9.4% (n = 15) and 8.8% (n = 14) of patients, respectively. Chronic lung disease and eczema each accounted for 6.3% (n = 10) of cases, while hyper-IgE syndrome and immunodeficiency disorder were each present in 5.0% (n = 8) of patients. Dermatitis was the least frequent diagnosis, observed in 4.4% (n = 7) of patients. Concerning serum IgE levels, the highest mean IgE was recorded in patients with hyper-IgE syndrome (3436 kIU/L), followed by immunodeficiency disorder (2234 kIU/L) and dermatitis (1771 kIU/L). The lowest mean IgE level was observed in patients with urticaria (902 kIU/L). The distribution of diagnoses and corresponding mean IgE levels is summarized in Table 5 and Figure 5.

**Table 5:** Distribution of diagnoses and mean IgE levels among patients AR: Allergic rhinitis; ABPA: Allergic bronchopulmonary aspergillosis

Diagnosis	n (%)	Mean IgE (kIU/L)
Asthma	32 (20.1)	1161
AR	29 (18.2)	1001
ABPA	24 (15.1)	1669
Bronchiectasis	15 (9.4)	1168
Urticaria	14 (8.8)	902
Chronic lung disease	10 (6.3)	1714
Eczema	10 (6.3)	1505
Hyper-IgE syndrome	8 (5.0)	3436
Immunodeficiency disorder	8 (5.0)	2234
Dermatitis	7 (4.4)	1771
Total	160 (100)	

Symptoms associated with major clinical diagnoses for elevated IgE levels

**Figure 6:**
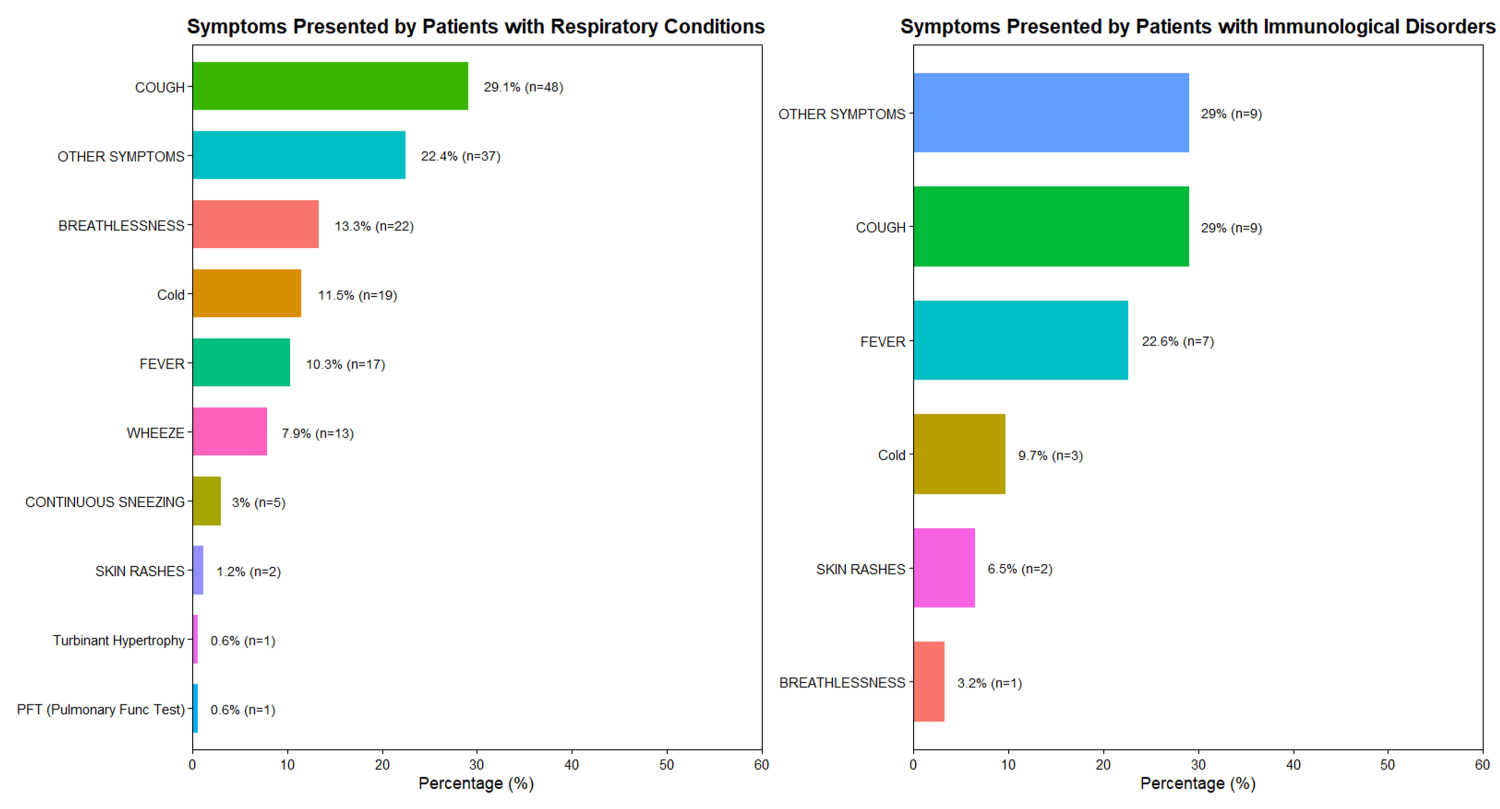
Symptoms associated with patients A: Respiratory conditions such as asthma, ABPA, AR, bronchiectasis, chronic lung disease, and respiratory distress; B: Immunological disorders such as hyper-IgE syndrome and immunodeficiency disorder ABPA: Allergic bronchopulmonary aspergillosis; AR: Allergic rhinitis

**Figure 7:**
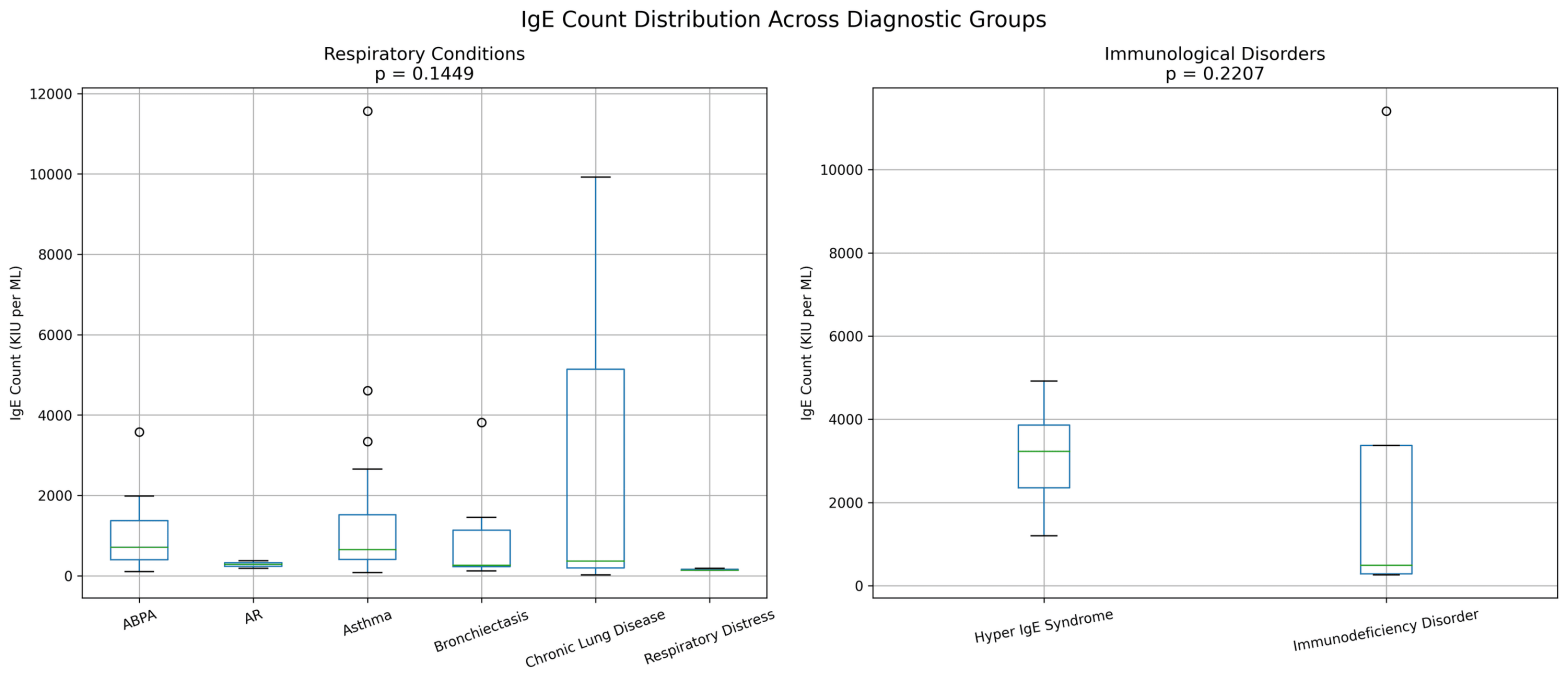
Distribution of serum IgE levels across respiratory and immunological diagnosis groups in paediatric patients ABPA: Allergic bronchopulmonary aspergillosis, AR: Allergic rhinitis

The number of patients was higher with respiratory conditions (n=165) compared to immunological disorders (n=32). The symptomatic profile was analyzed between the respiratory conditions and immunological disorders. Among respiratory patients, cough was the most frequently reported symptom (29.1%, n = 48), followed by other symptoms (22.4%, n = 37), breathlessness (13.3%, n = 22), cold (11.5%, n = 19), and fever (10.3%, n = 17). Less common symptoms in this group included wheezing (7.9%, n = 13), continuous sneezing (3.0%, n = 5), skin rashes (1.2%, n = 2), turbinate hypertrophy (0.6%, n = 1), and abnormal pulmonary function test findings (0.6%, n = 1). In patients with immunological disorders, other symptoms and cough were equally the most prevalent presentations (29.0% each, n = 9), followed by fever (22.6%, n = 7), cold (9.7%, n = 3), skin rashes (6.5%, n = 2), and breathlessness (3.2%, n = 1). Notably, wheezing and continuous sneezing were exclusive to the respiratory group, while fever was proportionally more prominent among immunological disorder patients. The distribution of symptoms across both groups is presented in Table 6 and Figures 6-7.

**Table 6:** Symptoms presented by patients with respiratory conditions and immunological disorders

Symptom	Patients with respiratory conditions, n (%)	Patients with immunological disorders, n (%)
Cough	48 (29.1)	9 (29.0)
Other symptoms	37 (22.4)	9 (29.0)
Breathlessness	22 (13.3)	1 (3.2)
Cold	19 (11.5)	3 (9.7)
Fever	17 (10.3)	7 (22.6)
Wheezing	13 (7.9)	—
Continuous sneezing	5 (3.0)	—
Skin rashes	2 (1.2)	2 (6.5)
Turbinate hypertrophy	1 (0.6)	—
Pulmonary function test (PFT)	1 (0.6)	—
Breathlessness	—	1 (3.2)

Respiratory Conditions

This boxplot compares IgE counts across different respiratory diagnoses, including asthma, ABPA, AR, bronchiectasis, chronic lung disease, and respiratory distress. Although some groups showed visibly different median IgE levels and variability, the Kruskal-Wallis test was not statistically significant (p = 0.1449). This suggests that IgE levels did not differ significantly between the respiratory diagnostic groups.

Immunological Disorders

This boxplot compares IgE counts between patients with hyper-IgE syndrome and immunodeficiency disorders. Patients with hyper-IgE syndrome generally demonstrated higher IgE values and wider variability; however, the Kruskal-Wallis test was not statistically significant (p = 0.2207). Therefore, no statistically significant difference in IgE levels was observed between the immunological disorder groups.

Correlation between elevated IgE levels and AEC

**Figure 8:**
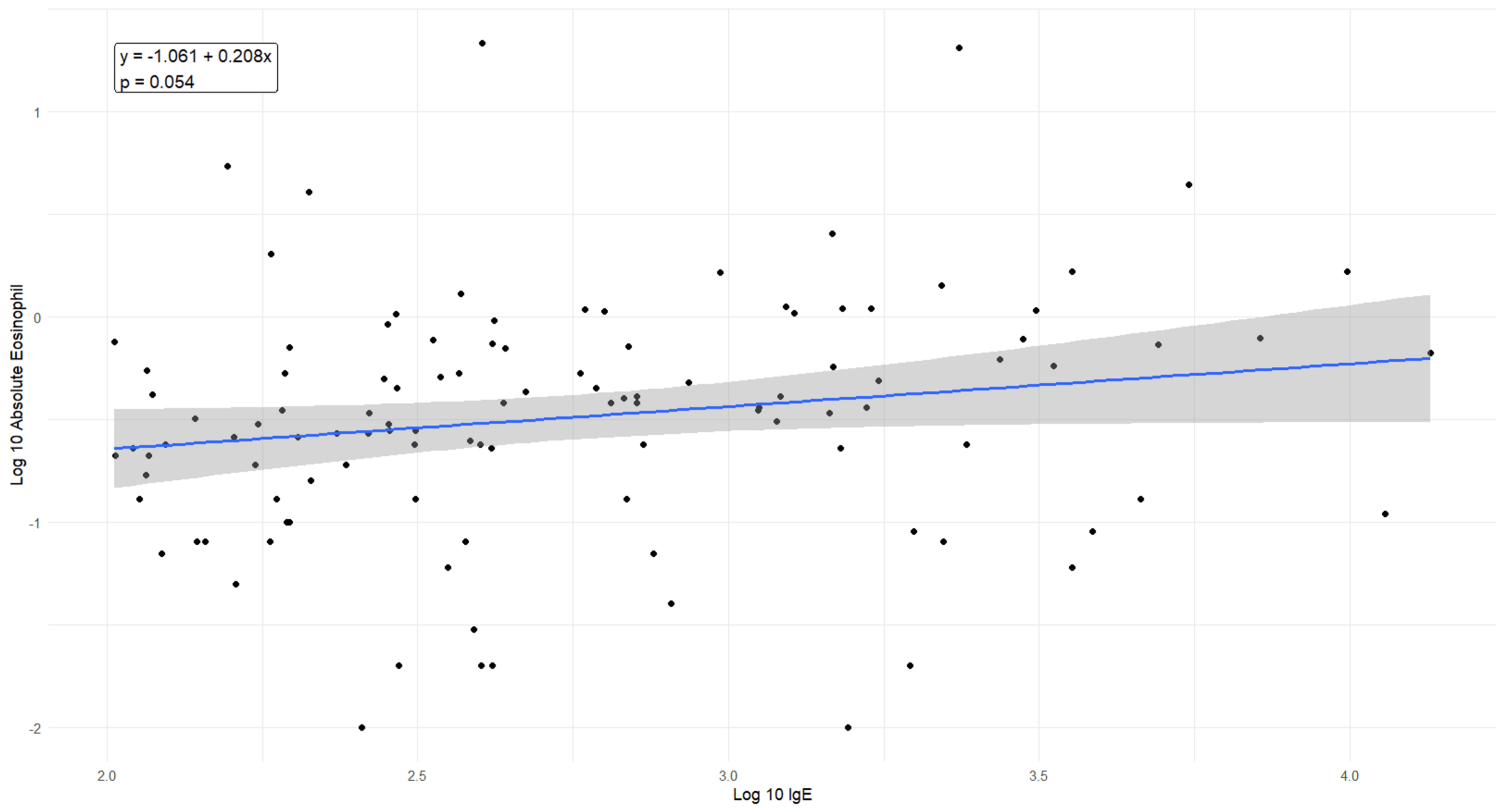
Spearman’s correlation between IgE levels and AEC (log scale) AEC: Absolute eosinophil count

With elevated IgE levels, there was a steady increase in AEC. So, Spearman’s rank correlation was analyzed. First, the Shapiro-Wilk test was performed to confirm whether the data are parametric or non-parametric. As the W values were very low (they should ideally be close to 1 for Pearson’s correlation), it was confirmed to be Spearman’s rank correlation. The Spearman’s rank correlation was calculated on a log scale, and the p-value was calculated as 0.008105 (<0.05), and the rho value as 0.2490196 (between 0.2 and 0.39). As the p-value was <0.05, the null hypothesis was rejected, and the data showed a significant correlation between elevated IgE levels and AEC. However, due to the lower rho value, the correlation is suggested to be a weak positive association (Figure 8).

## Discussion

The current study has shown a noteworthy increase with age in the percentage of measured elevated serum IgE [18], particularly in the 11-15-year-old age group (Figure 2), with statistically significant differences noted (p=0.0049). A greater number of males had elevated serum IgE than females; however, there was no significant association, as the male population was higher than the females in the study (Figure 3). The majority of elevated serum IgE levels measured were in the range of 100-500 kIU/L (Figure 4), indicating that elevated IgE is the most commonly found elevation related to most atopic disorders [19].

Of the total IgE-positive cases, the majority were seen in the pediatric OPD (Figure 5), supporting that the majority of cases presented were stable allergic conditions with less critically ill patients, which require ICU admission. Most frequently given diagnoses to patients with elevated IgE included asthma and AR (Figure 6), which also confirm the primary involvement of IgE in respiratory atopy [20]. Conversely, a few cases were diagnosed with hyper-IgE syndrome or other immunodeficiencies (i.e., with markedly elevated levels of IgE), indicating possible congenital immune deficiency disorders. [21]. Of the total cases in this study, those diagnosed with respiratory disorders had a much higher percentage of cough as a symptom compared to rash symptoms for skin disorders, suggesting an organ-specific distribution of IgE-mediated inflammation (Figure 6) [22]. For respiratory conditions, IgE levels varied across respiratory diagnoses, with higher values observed in asthma and ABPA cases; however, the differences were not statistically significant (p = 0.1449), and immunological disorders like hyper-IgE syndrome showed comparatively higher and more variable IgE levels than immunodeficiency disorders, though the difference was not statistically significant (p = 0.2207) (Figure 7). A significant positive correlation between elevated serum IgE and AEC was observed (Spearman's correlation), reinforcing (Figure 8) the involvement of eosinophilic inflammation as a factor in the pathogenesis of childhood allergic disease [23,24].

The treatment to help asthma was inhaled corticosteroids given by metered dose inhalers (MDI) [25], most often combined with bronchodilators, which would have likely worked to reduce the degree of inflammation in the airways. For AR there were intranasal corticosteroids, oral antihistamines, and leukotriene receptor antagonists that made up the majority of the medications prescribed to the patient [26,27]. Furthermore, the decreasing level of IgE levels demonstrates that the monitoring of total IgE levels on a serial basis provides an adjunct marker that could be used to monitor the therapeutic response to treatment in young children with atopic diseases [28].

The first limitation is that it is a cross-sectional study, so it is not possible to determine whether elevated IgE levels are causally related to any particular allergic disease. The patients studied were from one tertiary care hospital; therefore, limitations exist when generalizing these results outside of the sample studied. In addition, total IgE within normal ranges was not analyzed with the clinical conditions and without specifications regarding what allergens those IgE levels were derived from; thus, no direct correlation between IgE levels and the allergen that triggered the IgE could be established. Other variables that could have affected IgE levels (e.g., environmental exposures, parasites, and socioeconomic factors) were not examined in detail. 

## Conclusions

The results of the study suggest that total serum IgE measurement is clinically important in the evaluation of pediatric patients with allergic diseases. The IgE levels increased significantly with age and were generally higher in adolescents compared to younger age groups. Most cases with elevated IgE levels were associated with respiratory allergic diseases, particularly asthma and AR. Diagnosis-wise analysis further demonstrated variation in IgE distribution across respiratory and immunological conditions, with patients diagnosed with asthma, ABPA, and hyper-IgE syndrome showing comparatively higher IgE values and wider variability. Although statistical comparison between diagnostic groups did not demonstrate significant differences, the observed trends support the clinical relevance of IgE profiling in distinguishing patterns of allergic and immunological disease presentation. The weak positive correlation observed between IgE levels and AEC further supports the role of IgE in allergic inflammation. As this was a descriptive observational study, the findings primarily reflect the distribution, clinical patterns, and associations of IgE levels within the study population rather than establishing causation. Therefore, the results should be interpreted as demonstrating clinically relevant trends and diagnostic associations rather than definitive predictive relationships. The use of an ELFA-based assay for IgE estimation appeared to be a reliable and valid method for assessing and monitoring allergic disorders in pediatric patients.
